# Poikilodermatous Plaque-like Hemangioma: Case Presentation and Literature Review

**DOI:** 10.3390/dermatopathology11020015

**Published:** 2024-05-21

**Authors:** Pablo Díaz-Calvillo, Francisco Vílchez-Márquez, Francisco Manuel Ramos-Pleguezuelos, Salvador Arias-Santiago

**Affiliations:** 1 Department of Dermatology, Hospital Universitario Virgen de las Nieves, Avenida de Madrid 15, 18012 Granada, Spain; francisco.vilchez.marquez.sspa@juntadeandalucia.es (F.V.-M.); salvadorarias@ugr.es (S.A.-S.); 2 Instituto de Investigación Biosanitaria ibs.GRANADA, Avenida de Madrid 15, 18012 Granada, Spain; 3 Department of Pathology, Hospital Universitario Virgen de las Nieves, Avenida de las Fuerzas Armadas 2, 18012 Granada, Spain; patologiafmrp@gmail.com; 4 Department of Dermatology, University of Granada, Avenida de la Investigación 11, 18016 Granada, Spain

**Keywords:** poikilodermatous plaque-like hemangioma, hemangioma, vascular proliferation

## Abstract

Poikilodermatous plaque-like hemangioma (PPH) is a recently described clinical and pathological entity, with only 18 cases reported in the literature. Although uncommon, this benign condition presents consistent clinical and histological findings. We present a new case of PPH in an 81-year-old male and review the existing literature. The persistence over time and the need to distinguish PPH from more significant lesions underscore the importance of its clinical and pathological recognition.

## 1. Introduction

Poikilodermatous plaque-like hemangioma (PPH) is a recently described clinical-pathological entity, with only 18 reported cases in the literature [1,2,3]. PPH is characterized by consistent clinical and histological findings replicated across all cases. Clinically, it is characterized by a plaque-like lesion, atrophic and erythematous, commonly on the lower limbs of males over 50 years. Histologically, there is a diffuse proliferation of thin-walled vessels in the dermis, accompanied by epidermal changes. PPH tends to persist over time with indolent behavior, but data about long-term follow-up are scarce. Understanding PPH is crucial to differentiate it from more consequential lesions, thereby avoiding unnecessary procedures. Here, we present a new case of PPH and provide a comprehensive review of the available literature.

## 2. Case Report

An 81-year-old male presented with a lesion on the left buttock persisting for the last 2 years, displaying gradual growth. He did not associate it with any treatment, trauma, insect bites, or recent foreign travel. Occasional bleeding was reported, unaccompanied by other symptoms. The patient had a medical history of type 2 diabetes treated with sitagliptin, hyperuricemia treated with allopurinol, and atrial fibrillation managed with acenocoumarol. No recent changes in medication were noted.

**Figure 1:**
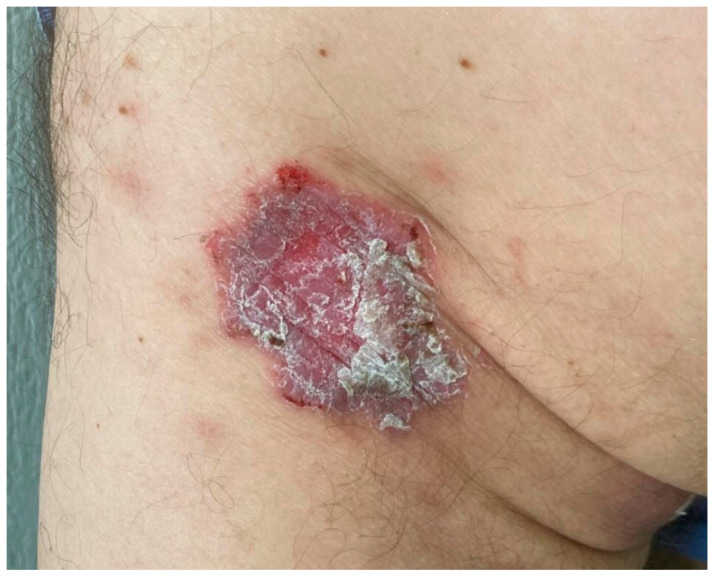
Erythematous scaly plaque on the left buttock. Note the hematic crust on the periphery of the lesion, more conspicuous in the upper area.

**Figure 2:**
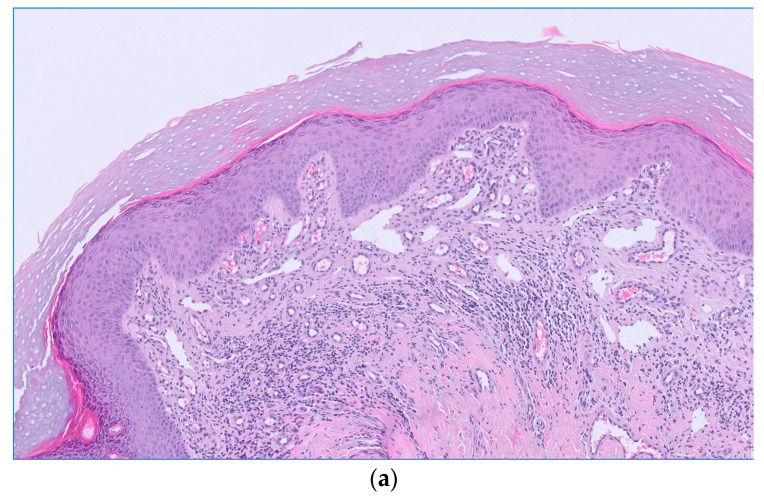

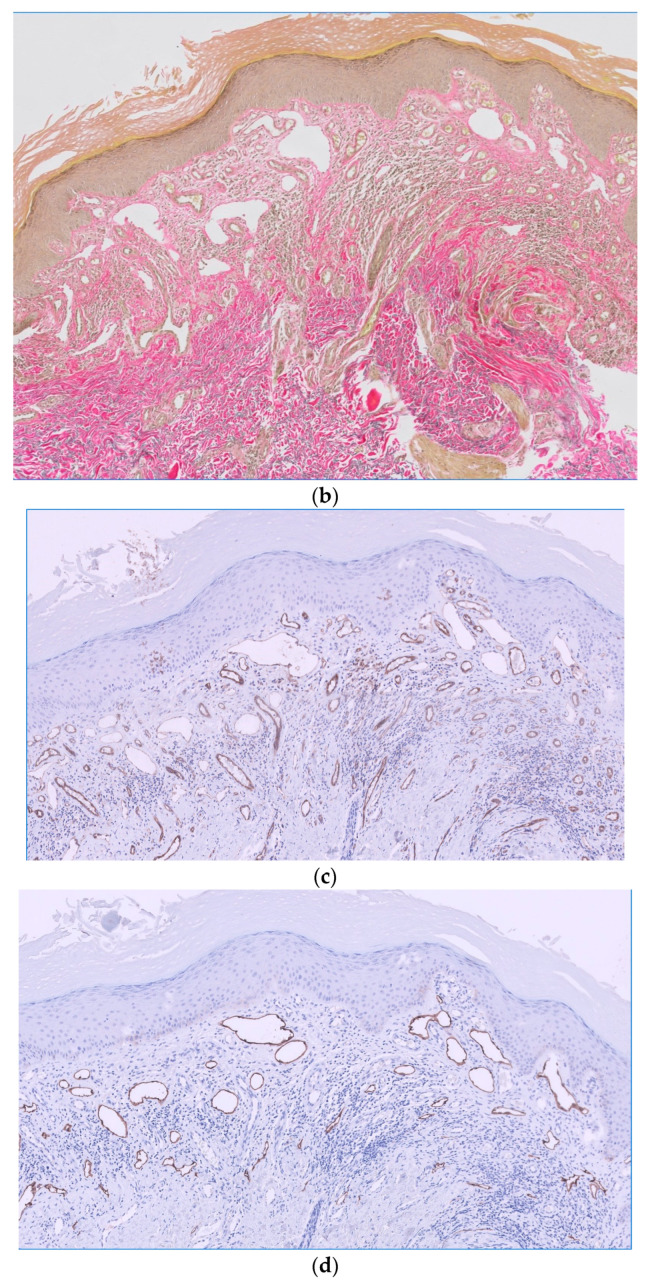
(**a**) Epidermis with orthokeratotic hyperkeratosis and irregular hyperplasia, proliferation of multiple thin-walled vessels, and vasodilation in superficial dermis, accompanied by a mild lymphohistiocytic perivascular infiltrate, haematoxylin–eosin, ×10. (**b**) Loss of dermal elastic fibers within the area of vascular proliferation, contrasting with adjacent normal dermis, Verhoeff-van Gieson stain, ×10. (**c**) Positive CD31 staining in blood vessels, immunohistochemistry, ×10. (**d**) Positive D2-40 staining in lymphatic vessels, immunohistochemistry, ×10.

Clinical examination revealed a poikilodermatous, infiltrated erythematous plaque measuring 6 × 4 cm on the left buttock with marked scaling (Figure 1). On dermoscopy, white scales, dotted vessels, and a red homogeneous background were observed, without any other specific findings. No other lesions were observed. An incisional biopsy, initially suspected as Bowen’s disease or superficial basal cell carcinoma, revealed orthokeratotic hyperkeratosis and a striking proliferation of thin-walled vascular channels and vasodilation in the superficial dermis, consistent with PPH (Figure 2a). A mild chronic lymphohistiocytic perivascular infiltrate lacking atypia was observed alongside the vascular proliferation. Verhoeff–van Gieson stain demonstrated loss of dermal elastic fibers within the area of vascular proliferation, contrasting with adjacent normal dermis (Figure 2b). Immune CD31 stain confirmed vascular origin (Figure 2c), and D2-40 was negative in primary vascular proliferation, but positive in background lymphatics (Figure 2d). No keratinocytic atypia or other suspicious features of malignancy were observed.

**Figure 3:**
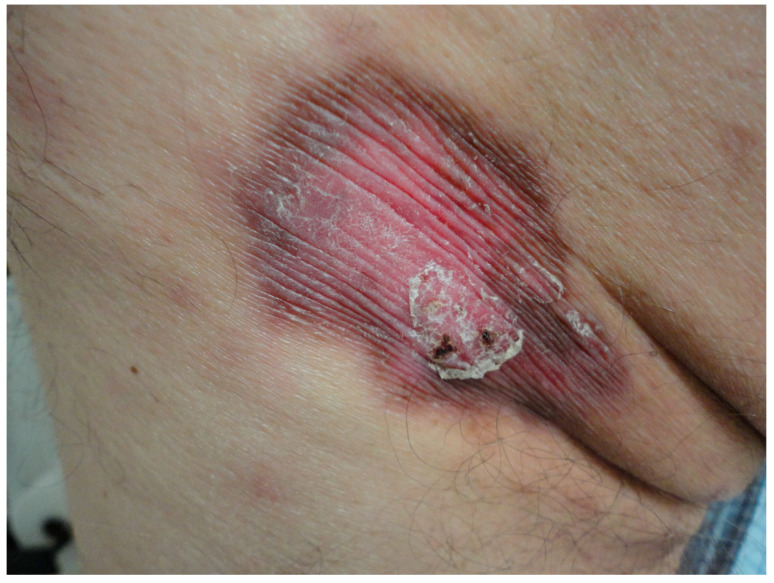
After 26 months barrier cream treatment. Persistence of the lesion, with a noticeable decrease in scaling and remnants of bleeding.

Barrier cream therapy was initiated. Over 26 months of follow-up, the lesion stabilized, with no more reported episodes of bleeding or associated symptoms. A reduction in erythema, scaling, and infiltration was evident (Figure 3).

## 3. Discussion

PPH was described in 2019 in a paper that collected 16 cases from United Kingdom patients [1]. Since then, two more cases have been reported [2,3]. The case we present would therefore be number 19. Men are mainly affected (17/19, 89.4%). The reported age range is from 58 to 90 years, with a mean age of 72.9 years (standard deviation 7.9) considering our case. 

Clinically, it presents as a solitary plaque ranging from 2 to 7 cm in diameter and erythematous to violaceous in color. Two out of the 19 patients reported had more than one lesion (2/19, 10.6%), resulting in a total of 22 lesions [1]. The lesions are mostly described as atrophic in appearance; however, they are not usually described as clinically scaly as in our patient, although epidermal changes, including hyperkeratosis and parakeratosis, are characteristic findings on histological examination. This scaly appearance prompted us to consider a diagnosis of squamous cell carcinoma in situ, despite the lesion being in a non-photoexposed area [4]. The most frequently described sites are the buttocks (5/22, 22.7%) and lower limbs (16/22, 72.7%), mainly the thigh (5/22, 22.7%), hip (4/22, 18.2%), and calf (3/22, 13.6%), although there is one case of involvement of the shoulder (1/22, 4.5%), which had two more lesions on the hip and buttocks [1,2,3]. The lesions described to date were asymptomatic and indolent, however, our case presented occasional episodes of bleeding, although without associated pain or pruritus. These episodes occurred in the first months, when the lesion was in the establishment phase and had more scaling. We think that this may be related to the significant scaling that was present, as the scale peeled off may have caused damage to the underlying skin, which could have led to the bleeding.

Only two cases preceding ours have documented dermoscopic features [1,2]. Hairpin-type vessels and scattered diffuse telangiectasias were described in these cases. In our case, we observed dotted vessels and a red homogeneous background, accompanied by desquamation. The varying conformations of vascular channels can result in different vessel forms on dermoscopy. However, with only a small number of dermoscopic findings available, it is challenging to generalize typical findings.

Histopathologically, PPH has constant features, as clearly described by Semkova et al. [1]. All cases described to date show a dermal vascular band-like proliferation of thin-walled vessels. The endothelial cells of these vessels lack atypical features and mitotic figures. Epidermal changes are also present. Hyperkeratosis is a constant finding and is also present in our case. Focal parakeratosis (3/19, 15.8%), acanthosis (13/19, 68.4%), hypergranulosis (3/19, 15.8%), spongiosis (5/19, 26.3%), lymphocyte exocytosis (6/19, 31.6%), and interphase vacuolar dermatitis (2/19, 10.5%) have also been described. In addition, dermal changes such as edema (13/19, 68.4%) and fibrosis (13/19, 68.4%) have been associated. In most cases, interstitial or perivascular lymphohistiocytic infiltrate was reported (17/19, 89.5%), as in our case, with no evidence of lymphocyte atypicality. Furthermore, elastic fibers are reduced or absent in the affected dermis.

The clinical differential diagnosis considered in our case included squamous cell carcinoma in situ and superficial basal cell carcinoma. The erythematous and scaly appearance, along with ulceration, initially raised suspicion of keratinocyte carcinoma [5]. However, the lesion’s occurrence in a non-sun-exposed area and histological findings, which did not reveal atypical keratinocytic cells, led us to rule out this diagnosis. Other differential diagnoses have been proposed in cases of PPH, with the most notable being mycosis fungoides (MF). Indeed, the clinical similarity and the importance of distinguishing between PPH and poikilodermic MF were the reasons for including “poikilodermic” in the hemangioma’s name [1]. MF typically presents as hyperpigmented patches or plaques with telangiectasias and an atrophic appearance, primarily affecting the trunk and flexural areas. Histologically, the presence of atypical lymphocytes in the epidermis is a classic finding of MF [6]. Fixed drug eruption is another dermatosis to consider, distinguished by its acute and recurrent nature and temporal relationship to a drug. Additionally, histologically, we observe vacuolar interface dermatitis, or dyskeratotic keratinocytes, with no proliferation of microvasculature playing a significant role [7]. The appearance on the lower limbs, combined with the purpuric aspect, may lead to confusion with pigmented purpuric dermatosis, primarily lichen aureus. These are typically asymptomatic or mildly pruritic, characterized histopathologically by hemorrhage extravasation and hemosiderin deposition, along with epidermal changes such as spongiosis, interface dermatitis, or exocytosis, without vascular proliferation [8]. The potential atrophic appearance of PPH also warrants consideration of other entities in the differential diagnosis, such as anetoderma or atrophoderma of Passini and Piernini, but the presence of vascular proliferation distinguishes PPH from these entities [2].

The etiology of PPH remains unknown, with proposed triggers such as arthropod bites or trauma unconfirmed. Most cases have been reported in Northern England, suggesting possible environmental or infectious factors [1]. Comorbidities have been documented in only 5 patients, including myelodysplastic syndrome, pulmonary sarcoidosis, hypertension, and type-2 diabetes, making it challenging to establish a clear association between HPP and any specific disease [1,2,3]. Frequent occurrence on lower limbs, particularly in pressure-prone areas, could imply a role of sustained trauma or pressure in the etiology. Likewise, the peripheral distribution of subcutaneous fat in women provides protection against pressure and trauma, and, therefore, this could be the reason why mainly men are affected [9]. In addition, the fact that older patients are mainly affected may be related to the increased sedentary lifestyle in this age group and, therefore, continuous pressure and loss of fat pad in pressure areas [10]. The observed reduction in erythema and bleeding with barrier creams in our case supports this hypothesis. 

However, factors other than purely mechanical ones must be involved. There are many older patients with sedentary lifestyles [11], but PPH is an extremely rare diagnosis. On the other hand, we think that this entity may be underdiagnosed, given the indolent course and the localization in generally covered and inconspicuous areas.

Being a recently described entity, there is limited information about the natural history of PPH, especially in the long term. The longest reported follow-up is 6 years. It exhibits an initial phase of gradual growth, accompanied in our case by significant hyperkeratosis and bleeding episodes, followed by a stabilization phase where lesion growth ceases [1,2,3]. Subsequently, the lesion persists, with no reported cases of regression. In our case, a reduction in erythema and scaling post-stabilization may be related to treatment or the natural course of the condition. Establishing hypotheses is challenging due to the scarcity of cases for comparison. 

The treatments used in the cases described are topical corticosteroids, oral corticosteroids, topical calcineurin inhibitors, and topical antifungals, none of which have been effective in changing the course of the disease [1,3]. The wait-and-see approach has been proposed as the most appropriate in this entity, given the indolent course [2]. Future research should explore emerging therapies and long-term outcomes.

## 4. Conclusions

We report a new case of PPH. This case underscores the importance of recognizing PPH, a rare but persistent benign entity. Understanding its clinical and histological features is crucial for differentiation from more concerning lesions. Continued reporting and analysis of cases are essential to enriching our understanding of this PPH and establishing effective treatments.

## Data Availability

The original contributions presented in the study are included in the article, further inquiries can be directed to the corresponding author.
